# Genome Sizes of Nine Insect Species Determined by Flow Cytometry and *k*-mer Analysis

**DOI:** 10.3389/fphys.2016.00569

**Published:** 2016-11-24

**Authors:** Kang He, Kejian Lin, Guirong Wang, Fei Li

**Affiliations:** ^1^Department of Entomology, College of Plant Protection, Nanjing Agricultural UniversityNanjing, China; ^2^State Key Laboratory for Biology of Plant Diseases and Insect Pests, Institute of Plant Protection, Chinese Academy of Agricultural SciencesBeijing, China; ^3^Ministry of Agriculture, Key Lab of Agricultural Entomology and Institute of Insect Sciences, Zhejiang UniversityHangzhou, China

**Keywords:** genome size, flow cytometry, *k*-mer analysis, sex difference, insect invasiveness

## Abstract

The flow cytometry method was used to estimate the genome sizes of nine agriculturally important insects, including two coleopterans, five Hemipterans, and two hymenopterans. Among which, the coleopteran *Lissorhoptrus oryzophilus* (Kuschel) had the largest genome of 981 Mb. The average genome size was 504 Mb, suggesting that insects have a moderate-size genome. Compared with the insects in other orders, hymenopterans had small genomes, which were averagely about ~200 Mb. We found that the genome sizes of four insect species were different between male and female, showing the organismal complexity of insects. The largest difference occurred in the coconut leaf beetle *Brontispa longissima* (Gestro). The male coconut leaf beetle had a 111 Mb larger genome than females, which might be due to the chromosome number difference between the sexes. The results indicated that insect invasiveness was not related to genome size. We also determined the genome sizes of the small brown planthopper *Laodelphax striatellus* (Fallén) and the parasitic wasp *Macrocentrus cingulum* (Brischke) using *k*-mer analysis with Illunima Solexa sequencing data. There were slight differences in the results from the two methods. *k*-mer analysis indicated that the genome size of *L. striatellus* was 500–700 Mb and that of *M*. *cingulum* was ~150 Mb. In all, the genome sizes information presented here should be helpful for designing the genome sequencing strategy when necessary.

## Introduction

Genome size is a species-specific characteristic that is not correlated with organismal complexity. This is often referred to as the C-value enigma or C-value paradox. The genome sizes of congeneric species are often significantly different and size can vary among individuals within a single species (Gregory, 2005; Tsutsui et al., 2008). Genome size differences among closely related species may be due to variation in the number of repetitive sequences (Boulesteix et al., 2006; Biémont, 2008). Genome size may be associated with a variety of physiological and environmental factors, but the forces influencing genome size remain unclear (Elizabeth Montiel et al., 2012).

Insects are the most diverse animal group with approximately 1,000,000 described species (Grimaldi and Engel, 2005; Tsutsui et al., 2008). A total of 948 genome size records from 793 insect species are recorded in the Animal Genome Size Database (Gregory, 2016). The genome sizes of prokaryotes are within a relatively narrow range, but eukaryote species vary by more than 200,000-fold (Gregory, 2001). Among the 14 orders of insects studied, the largest genome was found in the mountain grasshopper *Podisma pedestris* (1C-value is 16.93 pg). This is about 170-fold larger than the smallest genomes (0.1 pg) of *Psychoda cinerea, Coboldia fuscipes, Aphidius colemani*, and *Peristenus stygicus* (Guo et al., 2015) and it indicates that insect genome sizes can vary greatly among species from different orders.

Next generation sequencing technology has generated genome sequences of 195 insects (Robinson et al., 2011), including Anoplura, Blattodea, Coleoptera, Diptera, Ephemeroptera, Hemiptera, Isoptera, Odonata, Orthoptera, Phasmatodea, Strepsiptera, Thysanoptera, Trichoptera, Hymenoptera, and Lepidoptera (Yin et al., 2016). The insect gene number is not proportional to the genome size, indicating the complexity of insect genetics (Gregory, 2002; Hahn and Wray, 2002; Gregory et al., 2013; Elliott and Gregory, 2015b; Guo et al., 2015).

Knowledge of genome size is necessary for planning an insect genome sequencing project because this relates to assembly difficulty and costs. However, the genome sizes of many agriculturally important insects are unknown. We estimated the genome sizes of nine agricultural insects, including two planthoppers (small brown planthopper (SBPH) *Laodelphax striatellus* and white-backed brown planthopper (WBPH) *Sogatella furcifera*), two beetles (rice water weevil (RWW) *Lissorhoptrus oryzophilus* and coconut leaf beetle (CLB) *Brontispa longissima*), and five natural enemies of insect pests, including three mirids (*Tytthus chinensis, Cyrtorrhinus livdipennis*, and *Apolygus lucorum*), and two wasps (*Encarsia sophia* and *Macrocentrus cingulum*). The planthoppers, SBPH and WBPH, are notorious insect pests of rice, which are the main vectors of rice stripped virus, southern rice black-streaked dwarf virus, etc. The beetles, RWW, and CLB, are invasive insect pests of China, causing huge yield loss of rice or coconut, respectively. The three mirids and two wasps are widely used as the biological control agents in field. Considering the importance of these insects in agricultural production, we choose them to determine their genome sizes.

*Drosophila melanogaster* was used as a reference species. The genomes of the pea aphid *Acyrthosiphon pisum* and brown planthopper (BPH) *Nilaparvata lugens* have been reported, and these species were used as positive controls. We used flow cytometry to estimate the genome sizes of all nine insects. Then, we carried out *k*-mer analysis for *L. striatellus* and *M. cingulum* with ~50X sequencing coverage using the Illumina Solexa sequencing platform.

## Materials and methods

### Insects

Three kinds of rice planthoppers (*L. striatellus, N. lugens*, and *S. furcifera*) were collected in 2010 from a rice field in Nanjing, China. They were maintained in the laboratory on seedlings of japonica rice variety Wuyujing 3 (*Oryza sativa* L.) under a 16L:8D photoperiod at 26 ± 2°C with 65 ± 5% relative humidity (RH). The rice was grown in nutrient rich soil in a climate chamber. RWW (*L. oryzophilus*) were provided by Professor Mingxing Jiang at Zhejiang University and two kinds of mirid bugs (*C. livdipennis* and *T. chinensis*) were provided by Professor Zengrong Zhu at Zhejiang University. CLB (*Brontispa longissimi*) was obtained from Professor Zhengqiang Peng at the Chinese Academy of Tropical Agricultural Science in Hainan province, China. *E. sophia* was provided by Professor Fanghao Wan at the Chinese Academy of Agricultural Science and *M. cingulum* was provided by Professor Jian Hu at Sun Yet-Sen University. The *D. melanogaster* Canton-S strain was used as the external reference (Bennett et al., 2003; Tsoumani and Mathiopoulos, 2012). The fly culture was fed on a cornmeal-agar-molasses medium and maintained under a 12L:12D photoperiod at 25 ± 2°C and 60 ± 5% RH. *A. pisum* and *A. lucorum* were maintained in a laboratory at the Chinese Academy of Agricultural Science, Beijing, China.

### Sample preparation and flow cytometry

Samples were prepared using a standard procedure with slight modification (Galbraith et al., 1983; Brown et al., 2005; Dolezel et al., 2007). Insects were anesthetized using carbon dioxide for 20 s and the heads were dissected in an ice-cold plastic Petri dish. The heads were completely homogenized in 500 μL ice-cold Galbraith's Buffer (pH 7.0) containing 45 mM MgCl_2_, 20 mM MOPS (3-N-morpholinopropane sulfonic acid), 30 mM sodium citrate, and 0.1% (vol/vol) Triton X-100. The homogenate was washed slowly 2–3 times and filtered into a 1.5-mL Eppendorf tube using 38-μm nylon mesh. RNA was removed by adding RNase A (Takara, Japan) to the homogenate at a final concentration of 20 μg/ml and incubated at 25°C for 10 min. Then the solutions were centrifuged at 1000 g for 5 min. The precipitates were suspended with 400 μL phosphate buffer (pH 7.4) and stained with 50 μg/mL propidium iodide stock solution in darkness at 4°C for 10 min. The suspensions obtained in the final step were analyzed using the MoFlo™ XDP High Speed Cell Sorter and Analyzer (Beckman Coulter, CA, USA). The cell DNA content was measured using the fluorescent intensity of each sample exposed to a laser at 488-nm wavelength. The same parameter settings were applied to *D. melanogaster* samples. Summit Software (Beckman Coulter, CA, USA) was used to obtain the nuclei peaks (FL3-Log-Height or FL3-A). The genome sizes of the samples were calculated as follows:

$$\begin{array}{l} \text{Sample }1\text{C value}=\text{Reference }1\text{C}\\ \times \left(\frac{\text{sample }2\text{C mean peak position}}{\text{reference }2\text{C mean peak position}}\right), \end{array}$$

where reference 1C-value is the genome size of *D. melanogaster*, which is 176.4 megabase pairs (Mb) (1 pg = 978 Mb). For haploid and triploid cells, sample peaks of the 1C and 3C positions were used, and the sample 1C-value was obtained by multiplying by 2 and 2/3, respectively (Dolezel et al., 2007). All experiments were repeated at least for two times.

### *k*-mer analysis estimation of genome sizes

A paired-end library with an insert size of approximately 350 bp was constructed using the Illumina TruSeq Nano DNA (350) DNA sample preparation kit, following manufacturer instructions, and was sequenced using the Illumina HiSeqX system in Macrogen Inc., Korea. Quality control of raw sequence data was done using FastQC. The reads were filtered before assembly to ensure that a pair of paired-end reads had more than 90% of bases with quality ≥Q20. High-quality cleaned Illumina sequences were subjected to *k*-mer counting using JELLYFISH (Marçais and Kingsford, 2011) with the *k*-mer size set to 17. *k*-mer depth distribution was counted and the peak value of the depth distribution was identified. Since the short reads by the Illumina Solexa sequencing are randomly generated, the depth of the *k*-mer coverage should follow a Poisson distribution. So, the mean *k*-mer depth equals the peak value of the *k*-mer depth distribution. The genome size was calculated using the formula:

$$\begin{array}{l} \text{Genome size}=\text{total number of }k-\text{mer}/\text{peak value of }k-mer\\ \text{frequency distribution}. \end{array}$$

## Results

### Genome sizes estimation of nine insect species by flow cytometry

**Figure 1:**
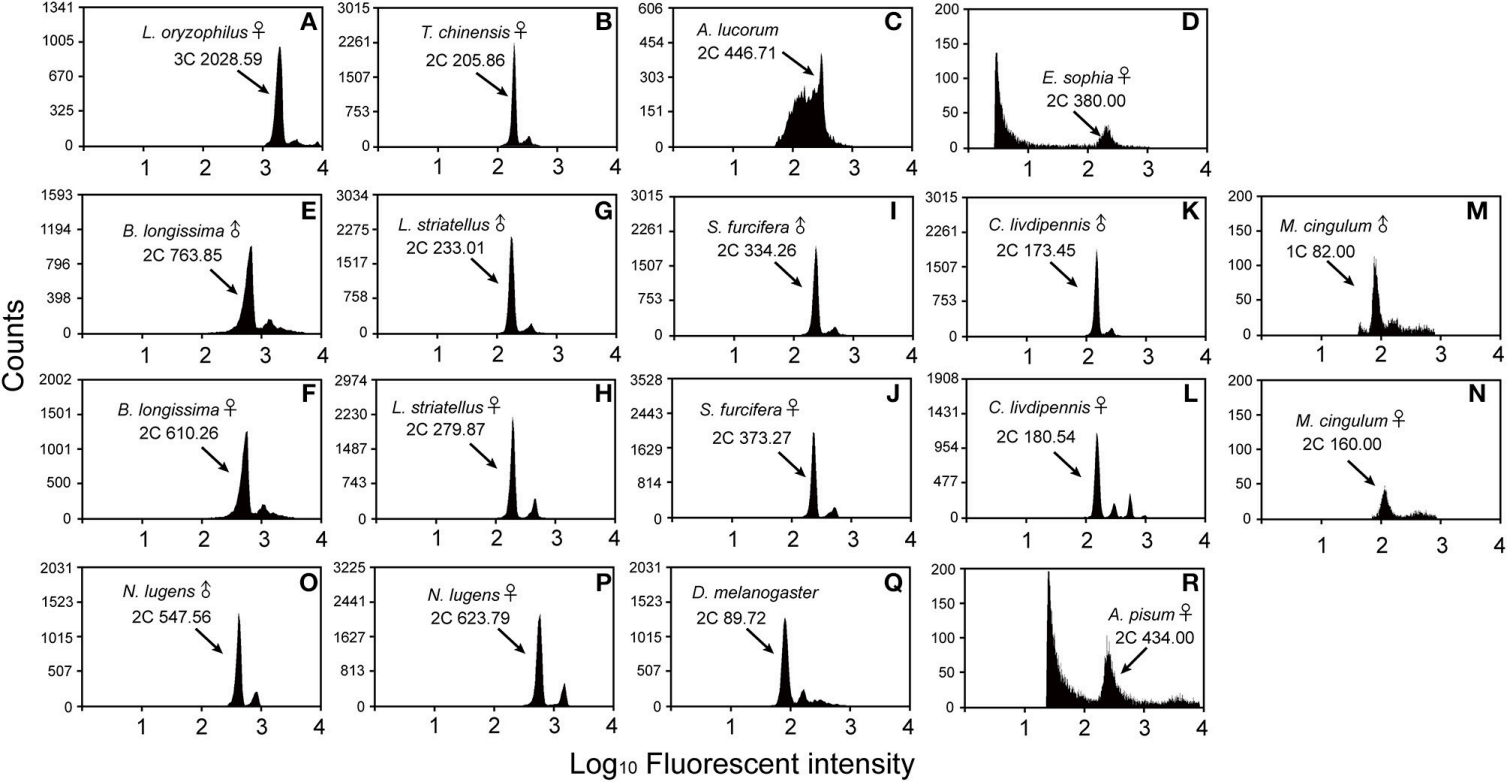
**Flow cytometry estimation of the genome size for the sex specific insects**. *D. melanogaster* was used as a reference standard and *N. lugens* and *A. pisum* were used as positive controls. The X-axis represents the relative fluorescence intensity of nuclei stained with propidium iodide in a nuclear suspension from head tissue. The Y-axis represents the number of nuclei. **(A)** *L. oryzophilus* females (981 Mb, 3C peak channel is 2028.59). **(B)** *T. chinensis* females (405 Mb, 2C peak channel is 205.86). **(C)** *A. lucorum* males (878 Mb, 2C peak channel is 446.71). **(D)** *E. sophia* females (372 Mb, 2C peak channel is 380.00). **(E)** *B. longissima* males (554 Mb, 2C peak channel is 763.58). **(F)** *B. longissima* females (443 Mb, 2C peak channel is 610.26). **(G)** *L. striatellus* males (458 Mb, 2C peak is channel 233.01). **(H)** *L. striatellus* females (555 Mb, 2C peak is channel 279.87). **(I)** *S.‘furcifera* males (657 Mb, 2C peak is channel 334.26). **(J)** *S. furcifera* females (734 Mb, 2C peak is channel 373.27). **(K)** *C. livdipennis* males (341 Mb, 2C peak is channel 173.45). **(L)** *C. livdipennis* females (354 Mb, 2C peak is channel 180.54). **(M)** *M. cingulum* males (161 Mb, 1C peak is channel 82.00). **(N)** *M. cingulum* females (157 Mb, 2C peak is channel 160.00). **(O)** Positive control *N. lugens* males (1077 Mb, 2C peak is channel 547.56). **(P)** Positive control *N. lugens* females (1226 mMb, 2C peak is channel 623.79). **(Q)** Mixed *D. melanogaster* males and females (176.4 Mb, 2C peak is channel 89.72). **(R)** Positive control *A. pisum* females (398 Mb, 2C peak is channel 434.00). The known genome size of positive control is 1137 Mb (*N. lugens* males), 1110 Mb (*N. lugens* females) and 464 Mb (*A. pisum* females), respectively.

The genome sizes of the nine insect species, estimated by flow cytometry, are presented in Table 1. *D. melanogaster* was the external reference and *N. lugens* and *A. pisum*, with published genomes, were positive controls. All experiments were repeated at least two times and the results indicated that all replicates had good reproducibility. The estimated genome sizes of *N. lugens* were 1130 Mb for male and 1110 Mb for female. *A. pisum* was estimated to be 460 Mb, which was consistent with genome assembly results (Richards et al., 2010; Xue et al., 2014). Among the nine insects, *L. oryzophilus* had the largest genome size (981 Mb, 1.003 pg), which was approximately two-fold that of other Coleoptera *B. longissimi* (554 Mb for male and 443 Mb for female). The genome sizes of the other eight insects (omitting *L. oryzophilus*) were <1 Gb (the nucleic content ≤1 pg) and the average genome size was 504 Mb. These data indicate that the insects studied had a moderate genome size. Both *S. furcifera* and *L. striatellus* had a smaller genome size than *N. lugens* (Figure 1). The mirids *C. livdipennis* and *T. chinensis* had a genome size around 400 Mb but *A. lucorum* had a genome size of 878 Mb. Hymenoptera tend to have smaller genome sizes than other orders. The genome sizes of wasps in this study were <200 Mb with the exception of *E. sophia*, which had a larger genome size of 372 Mb.

**Table 1:** **Genome sizes of nine insect species estimated by flow cytometry**.

**Order**	**Family**	**Species**	**Sex**	**1C-value (pg)**	**Genome size (Mb)**	**SE (Mb)**	***N***
Coleoptera	Curculionidae	*L. oryzophilus*	F	1.003	981	51	4
	Hispidae	*B. longissima*	M	0.566	554	5	3
			F	0.453	443	10	3
Hemiptera	Delphacidae	*L. striatellus*	M	0.468	458	12	3
			F	0.567	555	25	8
	*S. furcifera*		M	0.672	657	4	3
			F	0.751	734	8	3
	Miridae	*A. lucorum*	M	0.898	878	20	3
		*C. livdipennis*	M	0.349	341	2	3
			F	0.362	354	5	3
		*T. chinensis*	F	0.414	405	9	3
Hymenoptera	Aphelinidae	*E. sophia*	F	0.380	372	6	3
	Braconidae	*M. cingulum*	M	0.165	161	9	3
			F	0.161	157	6	3

*1 pg = 978 Mbp; F, Female; M, Male; N, number of individuals used; SE, standard error of the mean genome sizes*.

### Sex differences of genome sizes

Males and females of five species, *B. longissima, L. striatellus, S. furcifera, C. liydipennis*, and *M. cingulum*, were used to estimate genome size. Genome sizes were different in the males and females of four species except *C. liydipennis*. The sex differences of genome size were 111, 97, 77, and 4 Mb in *B. longissima, L. striatellus, S. furcifera*, and *M. cingulum*, respectively (Table 1, Figure 1). The largest difference between males and females was in *B. longissima*, while the smallest difference was in *M. cingulum*. The sex difference of genome size may have been due to variation in sex chromosome numbers. Male *L. striatellus* and *S. furcifera* have 29 chromosomes whereas female have 30 chromosomes (Kobayashi and Noda, 2007; Noda, 2009). The female hemipterans, *L. striatellus, S. furcifera*, and *C. liydipennis*, had larger genomes than the males. However, the genome sizes of male *B. longissima* and *M. cingulum* were larger than females. The sex related differences of genome size is a complex and unanswered scientific question.

### Genome sizes of alien invasive insects

Non-native, invasive species often cause damage to the environment, agriculture, economy, and humans. Among the nine insects studied, *L. oryzophilus* and *B. longissimi* are invasive insects. Both of these species cause significant economic losses in China. We investigated the relationship between species invasiveness and genome size. We collected genome size information on 18 invasive insects from the Animal Genome Size Database (http://www.genomesize.com). The genome sizes of invasive insects were compared with the average genome size of non-invasive species in the corresponding family. There was no distribution bias in the genome sizes of invasive insects (Table 2), suggesting that the invasiveness of an organism is unrelated to genome size.

**Table 2:** **Genome size comparison of 20 alien invasive insects in China**.

**Order**	**Family**	**Species**	**1C-value (pg)**	**Genome size (Mb)**	**1C-value of the corresponding family**	
					**Mean value (pg)**	**Range (pg)**
Blattodea	Blattidae	*Periplaneta americana* (Linnaeus)	**3.41**	**3335**	3.22	3.03–3.41
	Blattellidae	*Blattella germanica* (Linnaeus)	2.00	1956	–	–
Coleoptera	Chrysomelidae	*Callosobruchus analis* (Fabricius)	**0.98**	**958**	0.87	0.17–3.69
		*Callosobruchus maculatus* (Fabricius)	**1.26**	**1232**		
		*Acanthoscelides obtectus* (Say)	**0.98**	**958**		
		*Zabrotes subfasciatus* (Boheman)	0.76	743		
		*Leptinotarsa decemlineata* (Say)	0.46	450		
	Curculionidae	*Anthonomus grandis* (Boheman)	0.85	831	1.43	0.16–5.02
		*L. oryzophilus* (Kuschel)	1.09			1066
		*Hypothenemus hampei* (Ferrari)	0.16			156
	Dermestidae	*Trogoderma granarium* (Everts)	0.27	264	1.04	0.27–1.49
	Hispidae	*B. longissima* (Gestro)	0.45	440	–	–
Diptera	Cecidomyiidae	*Mayetiola destructor* (Say)	0.16	156	–	–
	Culicidae	*Aedes albopictus* (Skuse)	**1.66**	**1623**	0.96	0.23–1.90
Hemiptera	Aleyrodidae	*Bemisia tabaci* (Gennadius)	0.70	685	–	–
Hymenoptera	Formicidae	*Solenopsis invicta* (Buren)	**0.77**	**753**	0.36	0.18–0.77
	Tephritidae	*Ceratitis capitata* (Wiedemann)	0.60	587	0.72	0.33–0.99
Isoptera	Rhinotermitidae	*Coptotermes formosanus* (Shiraki)	0.93	910	1.02	0.93–1.07
Lepidoptera	Arctiidae	*Hyphantria cunea* (Drury)	0.66	645	0.70	0.46–1.13
Thysanoptera	Aeolothripidae	*Frankliniella occidentalis* (Pergande)	0.35	342	0.40	0.35–0.44

*The list of alien invasive insects was downloaded from China Agriculture Pest Information System. The genome size information of 20 species was downloaded from the Animal genome size database. The results indicate that half of the invasive insects have a smaller genome size compared with other insects in the same family. However, 30% of invasive insects have a larger genome size. “–” which implies that there is only one invasive species in the family. Bold number means the invasive species has a larger genome than the average size of species in the corresponding family*.

### *k*-mer analysis of *L. striatellus* and *M. cingulum* genome size

**Figure 2:**
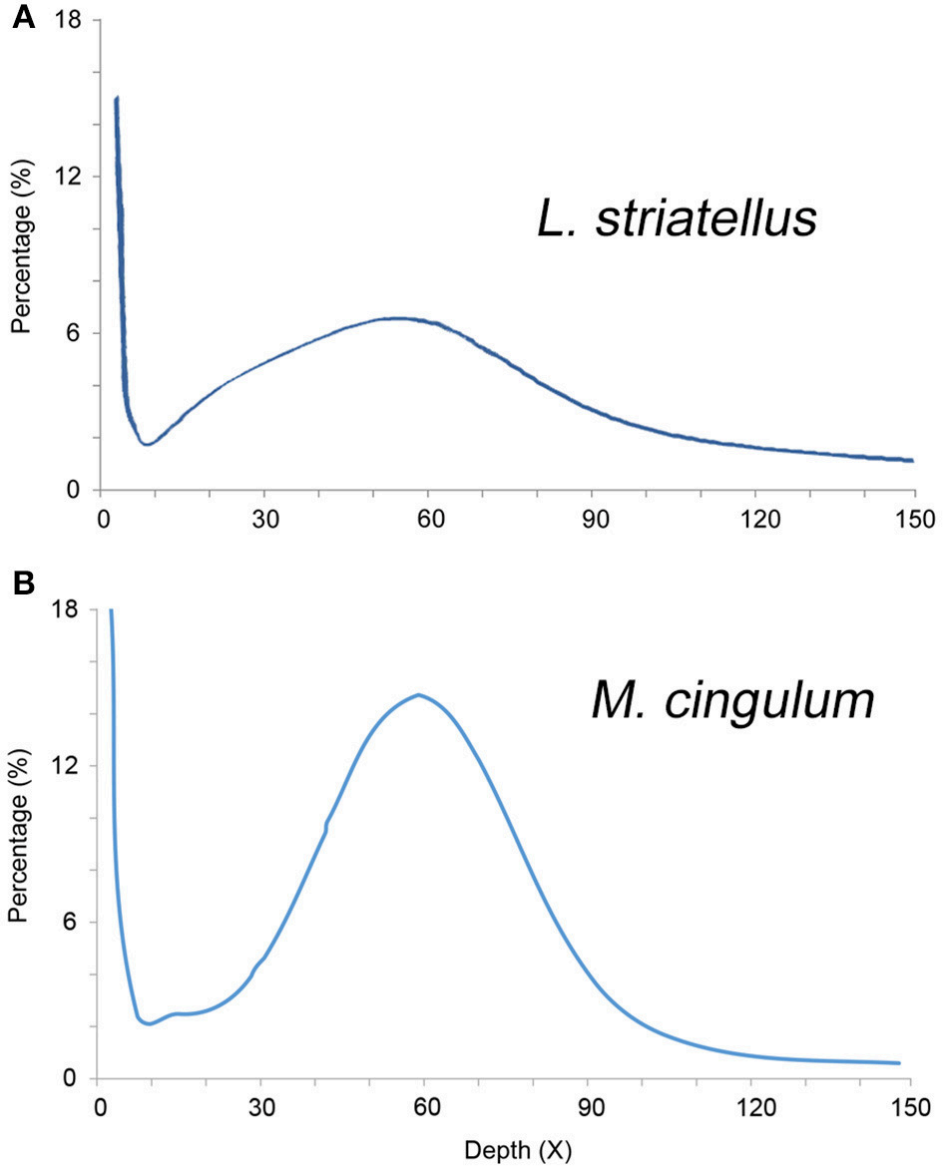
**17-mer frequency percentage distribution curve of sequencing reads of diploid ***L. striatellus*** (A)** and *M. cingulum* **(B)**. The X-axis represents the sequencing depth (X), and the Y-axis represents the proportion of specific *k*-mers to the total *k*-mer numbers with a giving sequencing depth. For *L. striatellus*, the 17-mer depth distribution graph shows a low level of heterozygosity and the genome size of *L. striatellus* was estimated to be 657 Mb. For *M. cingulum*, the *k*-mer depth distributions with a minor peak indicate a relatively low level of heterozygosity (0.4%) and the genome size was estimated to be 136 Mb.

The *k*-mer analysis was used to estimate the genome sizes of *L. striatellus* and *M. cingulum* using Illumina Solexa sequencing data. The 17-mer depth distributions showed a single peak, indicating that both insects had very low heterogeneity. Based on *k*-mer analysis, the genome size of *L. striatellus* was 657 Mb and that of *M. cingulum* was 136 Mb (Figure 2). These values were slightly different from estimates made using flow cytometry. This difference might be due to the variability of the two methods. Another possible reason was that different samples were used for the *k*-mer analysis. If this is the case, the genome size differences between different samples require further investigation. Regardless, it still can be concluded that *L. striatellus* had a moderate-size genome of 500–700 Mb while *M. cingulum* had a small-size genome of ~150 Mb. The exact genome size requires confirmation by additional genome-sequencing.

## Discussion

Three planthoppers, *N. lugens, S. furcifera*, and *L. striatellus*, belong to the Delphacidae family. These planthoppers are the most destructive rice insect pests in Asia (Noda, 2009; Yin et al., 2014). The chromosome numbers of all 3 are 30 except male *S. furcifera* and male *L. striatellus*, which have 29 chromosomes (28+XO) (Kobayashi and Noda, 2007; Noda, 2009). This might explain the fact that male *S. furcifera* and male *L. striatellus* have a significantly smaller genome size than females. The difference between male and female is a very interesting question and is worthy of further investigation.

*L. oryzophilus* and *B. longissima* are quarantined invasive beetles that cause huge losses of rice or coconut in China (Chen et al., 2005; Ju et al., 2005; Saito et al., 2005; Lu et al., 2008). *L. oryzophilus* evolved asexual reproduction by parthenogenesis after invading into the Asian rice production region and populations grew rapidly. The triploid female of *L. oryzophilus* has 33 chromosomes (Takenouchi, 1978; Saito et al., 2005). We analyzed the genome size distribution of 20 invasive insects and did not found any relationship between invasiveness and genome size, suggesting that insect invasiveness might be associated with other genetic factors but not the genome size.

All nine insects varied significantly in genome size. The genome sizes of 948 insects have previously been measured. Insect genome sizes are highly variable, even within the same family (data not shown). Differences in genome size may arise through accumulation of transposable elements (TEs) and expansion of intron size (Zhang and Edwards, 2012). However, TE diversity does not increase with genome size when it exceeds about 500 Mb (Elliott and Gregory, 2015a). Other, less obvious, reasons affect genome size variation in eukaryotes. The variance of genome sizes among close species remains a mystery. Large scale analysis of all known 948 insect genome sizes may provide insight into possible mechanisms. We observed intraspecific genome size differences between males and females. The reason for this difference and its influence on sex-specific or sex-biased gene expression is still unclear and requires further clarification.

The genomic size of an organism provides useful information. We determined the genome sizes of nine agriculturally important insects, and this information would justify additional efforts to sequence their genomes. By use of flow cytometry and *k*-mer analysis results, we have designed a genome-sequencing strategy and successfully obtained a preliminary genome of *M. cingulum* (data unpublished). These data indicate that *k*-mer analysis is more accurate for estimating genome size.

## Author contributions

KH carried out all experiments, analyzed the data and drafted the manuscript. KL and GW participated in the discussion, experiment design. FL conceived and designed all experiments, analyzed the data and wrote the manuscript.

## Funding

This work was in partial supported by the National Key Research and Development Program (2016YFC1200600) and the science and technology research project of the Ministry of Education, China (V201308).

### Conflict of interest statement

The authors declare that the research was conducted in the absence of any commercial or financial relationships that could be construed as a potential conflict of interest.
